# MEIS2 regulates endothelial to hematopoietic transition of human embryonic stem cells by targeting TAL1

**DOI:** 10.1186/s13287-018-1074-z

**Published:** 2018-12-07

**Authors:** Mengge Wang, Hongtao Wang, Yuqi Wen, Xiaoyuan Chen, Xin Liu, Jie Gao, Pei Su, Yuanfu Xu, Wen Zhou, Lihong Shi, Jiaxi Zhou

**Affiliations:** 1 State Key Laboratory of Experimental Hematology, Institute of Hematology and Blood Diseases Hospital, Tianjin, 300020 China; 2 Center for Stem Cell Medicine, Chinese Academy of Medical Sciences and Department of Stem Cells and Regenerative Medicine, Peking Union Medical College, Tianjin, 300020 China; 3 School of Basic Medical Science and Cancer Research Institute, Central South University, Changsha, 410013 China

**Keywords:** MEIS2, Hematopoiesis, EHT

## Abstract

**Background:**

Despite considerable progress in the development of methods for hematopoietic differentiation, efficient generation of transplantable hematopoietic stem cells (HSCs) and other genuine functional blood cells from human embryonic stem cells (hESCs) is still unsuccessful. Therefore, a better understanding of the molecular mechanism underlying hematopoietic differentiation of hESCs is highly demanded.

**Methods:**

In this study, by using whole-genome gene profiling, we identified Myeloid Ectopic Viral Integration Site 2 homolog (MEIS2) as a potential regulator of hESC early hematopoietic differentiation. We deleted MEIS2 gene in hESCs using the CRISPR/CAS9 technology and induced them to hematopoietic differentiation, megakaryocytic differentiation.

**Results:**

In this study, we found that MEIS2 deletion impairs early hematopoietic differentiation from hESCs. Furthermore, MEIS2 deletion suppresses hemogenic endothelial specification and endothelial to hematopoietic transition (EHT), leading to the impairment of hematopoietic differentiation. Mechanistically, TAL1 acts as a downstream gene mediating the function of MEIS2 during early hematopoiesis. Interestingly, unlike MEIS1, MEIS2 deletion exerts minimal effects on megakaryocytic differentiation and platelet generation from hESCs.

**Conclusions:**

Our findings advance the understanding of human hematopoietic development and may provide new insights for large-scale generation of functional blood cells for clinical applications.

**Electronic supplementary material:**

The online version of this article (10.1186/s13287-018-1074-z) contains supplementary material, which is available to authorized users.

## Background

Hematopoietic stem cell transplantation remains to be the only effective approach to treat patients with hematopoietic disorders, such as leukemia, lymphoma, and sickle cell anemia [1]. However, the scarcity of HLA-matched HSCs severely limits the clinical applications [2]. Thus, there is a strong need to find alternative sources to generate HSCs in vitro to meet the clinical demand. Human pluripotent stem cells (hPSCs), including human embryonic stem cells (hESCs) and human induced pluripotent stem cells (hiPSCs), represent a promising source for the generation of HSCs due to its self-renewal ability and multi-lineage differentiation potential [3]. Since the hematopoietic differentiation capacity of hESCs was first confirmed in 2009 by Dr. James A. Thomson’s group [4], significant advantages have been made in the field of hematopoietic differentiation from hPSCs, including the establishment of multiple hematopoietic differentiation protocols and the generation of functional blood cells [5]. However, derivation of bona fide HSCs with robust multi-lineage engraftment potential from hPSCs remains a major challenge [6]. A thorough understanding of the molecular mechanism underlining hematopoietic fate decision is essential for the development of novel approaches to direct hPSCs to undergo the HSC fate.

Hematopoietic differentiation from hPSCs goes through four main stages: the emergence of mesoderm, the specification of lateral mesoderm, the generation of hemogenic endothelial cells (HEPs), and, finally, the endothelial to hematopoietic transition (EHT), with different molecular markers appearing sequentially during hematopoiesis [7]. EHT represents a key event in hematopoietic fate commitment and is tightly regulated by extrinsic signals and intrinsic factors. It has been shown that NOTCH signaling promotes EHT, while TGFβ signaling exerts an inhibitory effect, and manipulation of these two signaling pathways markedly enhances the generation of hematopoietic cells from hPSCs [8–10]. In addition, accumulating evidence demonstrates that transcriptional factors play critical roles in hematopoietic transition from endothelium. Runx1 is considered as an essential regulator during EHT in the aorta-gonad-mesonephros (AGM) region, and enforced expression of RUNX1a enhances hematopoietic lineage commitment of hPSCs [11]. GATA2 deletion impairs the generation of hematopoietic cells from hPSCs by suppressing EHT [12]. TAL1 plays an important role in both HEP generation and hematopoietic transition from endothelium during hematopoiesis of hPSCs [13]. HES1 functions downstream of NOTCH signaling and induces hematopoietic differentiation of hPSCs by refraining the endothelial commitment of HEPs [10]. In contrast, HOXA3 and SOX7 play suppressive roles in EHT by maintaining the endothelial property of HEPs [14, 15]. Consistent with the vital role of transcriptional factors in the process of hematopoietic fate decision, an increasing number of studies have shown that blood cells can be directly induced from hPSCs or somatic cells through ectopic expression of transcription factors. For instance, ETV2/GATA2 and GATA2/TAL1 can directly convert hPSCs to blood cells with pan-myeloid or erythro-megakaryocytic potential, respectively [16]. Hematopoietic cells with engraftment potential can be reprogrammed from human endothelial cells through expression of four transcription factors—FOSB, GFI1, RUNX1, and SPI1—in the vascular-niche microenvironment [17]. Thus, transcription factors clearly play critical roles in hematopoietic specification. Therefore, identification of novel transcription factors in directing hematopoietic differentiation, especially at the stage of EHT, is essential for a better understanding of hematopoiesis and consequently the de novo generation of clinical-grade HSCs.

MEIS (myeloid ectopic insertion site) homeodomain transcription factors belong to the TALE (three-amino acid loop extension) superfamily and consist of three homologs MEIS1, MEIS2, and MEIS3 in human [18]. MEIS proteins commonly function as HOX co-factors and regulate target gene transcription by directly binding to PBX proteins [19]. Meis2 is widely expressed in mice and participates in the development of multiple organs, such as the brain, limb, lens, retina, and heart [20]. Consistent with the function of Meis2 in mice, the patients carrying MEIS2 mutations present developmental abnormalities, including intellectual disability (ID), cleft palate, and defects in heart development [21–23]. Recent studies reported the critical role of MEIS2 in normal and malignant hematopoiesis. MEIS2 is highly expressed in AML1-ETO (AE)-positive AML and promotes leukemogenesis by directly binding to AML1-ETO and thus impairing its DNA binding ability [24]. A recent study revealed that Meis2 is substantially upregulated in MN1 leukemic cells and mediates MN1-induced leukemic activity [25]. Meis2-deficient mice present a small fetal liver size and display embryonic lethality between E13.5 and E14.5 [20], suggesting that Meis2 may influence embryonic hematopoiesis. In accordance with these observations, Meis2 overexpression promotes hematopoietic induction from mouse embryonic stem (ES) cells [26]. However, the stage at which Meis2 regulates hematopoietic differentiation and the underlying mechanism remain undefined. In addition, whether MEIS2 has any roles in early hematopoietic differentiation in humans is still unknown. Like Meis1, mice lacking Meis2 show severe hemorrhaging [20], suggesting that Meis2 might also influence embryonic megakaryopoiesis just like Meis1. Consistently, enforced expression of Meis2 in mouse embryonic stem cells skews hematopoietic differentiation to megakaryocytic progenitor while suppressing erythroid differentiation [26]. We recently reported that MEIS1 deletion severely impairs hPSC megakaryocytic differentiation and almost completely blocks platelet generation [27]. Thus, in this study, we also explored whether MEIS2 plays similar roles in human megakaryopoiesis and thrombopoiesis. By taking advantage of genome-wide transcriptome sequencing and the CRISPR/CAS9 technology, we identified MEIS2 as a vital regulator of hESC hematopoietic differentiation. We also found that MEIS2 regulates early hematopoietic differentiation by enhancing HEP specification and EHT and does so by targeting TAL1. Together, we identify MEIS2 as a novel regulator of early human hematopoietic differentiation and provide new mechanistic insights for human hematopoiesis. Our results may facilitate the development of new strategies for large-scale generation of functional blood cells.

## Methods

### hESC hematopoietic differentiation in chemical defined system

The chemical defined system (CDS) was performed as previously described with some modifications to induce hESC hematopoietic differentiation. Seventy to eighty percent confluent hESCs were dissected into single cells using 1 mg/ml Accutase (Gibco) and plated on growth factors reduced (GFR) Matrigel-coated dishes at a density of 3.5 × 104 cells/well (12-well plate) in mTeSR1 medium containing 10 μM Y27632 (Calbiochem). Twenty-four hours later, hESCs were induced into stepwise differentiation. First, cells were cultured in Custom mTeSR1 medium (Stem Cell Technologies) supplemented with 50 ng/ml ActivinA (Peprotech) and 40 ng/ml BMP4 (Peprotech) for 2 days. Second, cells were incubated with Custom mTeSR1 medium supplemented with 40 ng/ml VEGF (Peprotech) and 50 ng/ml bFGF (Peprotech) for 2 days. Third, cells were incubated with Custom mTeSR1 medium supplemented with 40 ng/ml VEGF, 50 ng/ml bFGF, and 20 μM SB 431542 (STEMGENT) for 3 days. Finally, differentiated cells were dissociated and seeded into low-attachment 24-well plate and cultured for 6 days in Custom mTeSR1 medium containing 20 ng/ml SCF (Peprotech), 50 ng/ml TPO (Peprotech), 20 ng/ml IL3 (Peprotech), 1 mM glutamax (Gibco), 2% B27 (Gibco), 0.1 mM MTG (Sigma-Aldrich), 1% ITS (Gibco), 1% NAA (Gibco), and 1% penicillin/streptomycin. Fresh medium was changed every 2 days.

### hESC megakaryocytic differentiation

Megakaryocytic differentiation based on mAGM-S3 co-culture system was carried out as previously described [27]. mAGM-S3 cells were cultured to form an overgrown monolayer and treatment of 5 ng/ml mitomycin C (Sigma-Aldrich) in 37 °C for 2 h before co-culture with hESCs. hESCs were prepared as small aggregates containing about 300 cells and suspended in mTESR1 medium. The small aggregates were re-plated on mAGM-S3 cells at a density of 15–20 aggregates/well (12-well plate). On the next day, hematopoietic induction medium was used to replace mTESR1; detailed information can be found in a previous study [27]. After 12-day culture, cobblestone cells derived from hematopoietic differentiation of hESCs were mechanically detached and seeded on mitomycin C-treated mAGM-S3 stromal cells in hematopoietic differentiation medium with the addition of Y-27632 (10 μM), TPO (50 ng/mL), SCF (20 ng/mL), IL-3 (20 ng/mL), IL-6 (10 ng/mL), and IL-11 (20 ng/mL). The medium was changed every 3 days.

### Establishing MEIS2 knockout hESC lines using CRISPR/CAS9 technology

Single-guide RNA (sgRNA) targeting human MEIS2 gene was designed using the CRISPR Design Tool (http://tools.genome-engineering.org) [28]. The sequence of corresponding oligonucleotide was cloned into lentiviral vector CRISPR-Cas9-Lenti-V2 (Addgene). Sequence-verified plasmids were denominated as Lenti-V2-MEIS2-E3G3. The genome editing efficacy of constructed plasmid was detected using surveyor in 293 T cells, and after verification, the vector was used for generating MEIS2 deletion hESC lines. To obtain MEIS2-deleted hESC lines, lentivirus containing MEIS2-E3G3 was infected into H1 hESCs, and hESCs were subsequently selected with puromycin (1 μg/ml, Sigma). After assessing the genome editing efficacy, cells were dissociated into single cells using Accutase (Gibco). Small colonies derived from individual cells were picked and expanded. Western blotting assay was performed to identify MEIS2 knockout hESC lines. hESC lines with anomalous expression of MEIS2 protein were identified by gene sequencing analysis. The sequence of primer used for surveyor assay was list as below, F: 5′-TATTTGTCGGGCTGCAGTGG-3′, R: 5′-TGTTCAAGTAGCTGGAGGCG-3′.

### RNA-seq and bioinformatics analysis

RNA-seq analysis was performed by BGI Company (BGI, Shenzhen, China) as previously described [27]. Heatmap was generated using HemI heatmap illustrator software (GPS) or R language. The RNA-seq data are available at Gene Expression Omnibus (GEO) (accession number: GSE115979).

### Statistical analysis

Student’s *t* test was used to evaluate the differences between two groups. Differences were defined when *P* value was less than 0.05. Statistical analyses were performed utilizing the GraphPad Prism software. Data are presented as the mean ± SD.

## Results

### MEIS2 is a potential regulator of early human hematopoietic differentiation

**Fig. 1:**
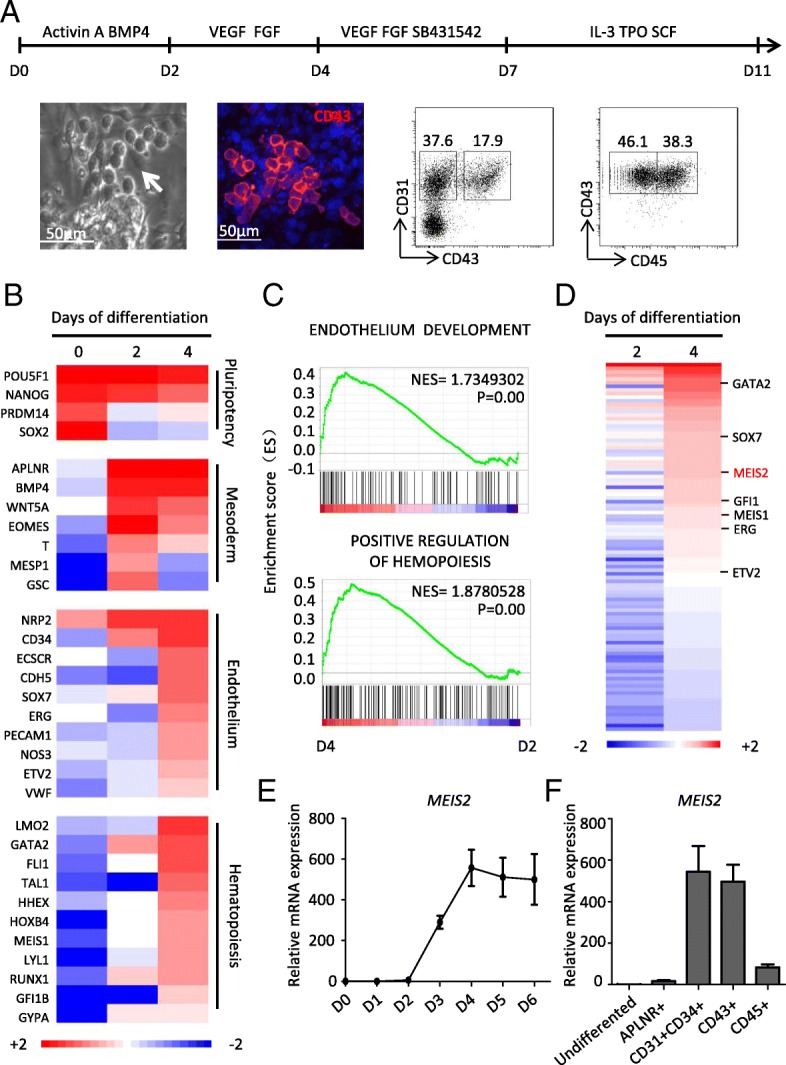
MEIS2 is a potential regulator of hESC early hematopoietic differentiation. **a** Schematic overview of chemical defined system to induce HPCs from hESCs (top). Hematopoietic cells were observed with immunofluorescence studies and flow cytometry (bottom). **b** Heatmap of transcriptional factors associated with pluripotency, mesoderm, endothelium, and hematopoiesis during early hematopoietic differentiation from hESCs. Analysis was performed by samples harvested at day 0, day 2, and day 4 of differentiation. **c** GSEA of hematopoietic development (day 2 vs day 4) in “endothelium development” and “positive regulation of hematopoiesis.” **d** Heatmap of transcription factors increased from day 2 to day 4 of hematopoietic differentiation (day 4 vs day 2, fold change > 2). **e** Dynamic analysis of MEIS2 expression with real-time PCR during hematopoietic differentiation of hESCs. Relative expression is normalized by undifferentiated hESCs. Data are shown as means ± SEM (*n* = 3). **f** Real-time PCR analysis of MEIS2 expression in undifferentiated hESCs, mesoderm (APLNR+), HEPs (CD31 + CD34+), and HPCs (CD43+ and CD45+) derived from hESCs. Relative expression was normalized by using undifferentiated hESCs. Data are shown as means ± SEM (*n* = 3)

We previously established a chemically defined hematopoietic differentiation system (CDS), which recapitulates embryonic hematopoiesis and allows the cells to go through the stages of mesoderm induction, mesoderm lateralization, hemogenic endothelium progenitor (HEP) specification, and endothelial to hematopoietic transition (EHT) [27, 29] (Fig. 1a). By taking advantage of this model, we subsequently identified MEIS1 as a crucial regulator of HEP specification [27]. In attempts to further our understanding of the regulators implicated in early human hematopoietic differentiation, we carried out an in-depth bioinformatics analysis of our previous data of genome-wide RNA-seq. As expected, the pluripotency-related genes were gradually downregulated during the hematopoietic induction process. In comparison with undifferentiated cells, the expression of mesoderm-associated genes increased significantly in cells at day 2, whereas endothelium and hematopoiesis-associated genes were profoundly upregulated at day 4 (Fig. 1b). Gene Set Enrichment Analysis (GSEA) further confirmed the enrichment of endothelium and hematopoiesis-associated genes in the differentiated cells at day 4 (Fig. 1c). These results suggested that the genetic program for hematopoiesis is activated at the stage of HEP specification from mesoderm cells, thus prompting us to screen potential key regulators of this process. As shown in Fig. 1d, 103 genes showed a sharp upregulation (day 4 vs day 2, greater than twofold; false discovery rate (FDR) < 0.01). Interestingly, we identified several genes known to be critical for human endothelium specification (SOX7, ERG, and ETV2) and hematopoiesis (GATA2, GFI1, and MEIS1), thereby verifying the screening strategy. Among those, MEIS2 was especially interesting because Meis2-deficient mice exhibited severe defects in hematopoiesis. Consistent with the data from RNA-Seq, MEIS2 expression began to increase dramatically at day 2 of differentiation, peaked at day 4, and maintained high levels of expression afterwards, as assessed with real-time PCR analysis (Fig. 1e). To further confirm the results, we determined the expression of MEIS2 in CD31^+^CD34^+^ HEPs and CD43^+^ hematopoietic cells and found that MEIS2 was markedly upregulated in these cells when compared with undifferentiated cells (Fig. 1f). Thus, MEIS2 expression increases during early human hematopoietic differentiation.

### MEIS2 deletion impairs early hematopoietic differentiation

**Fig. 2:**
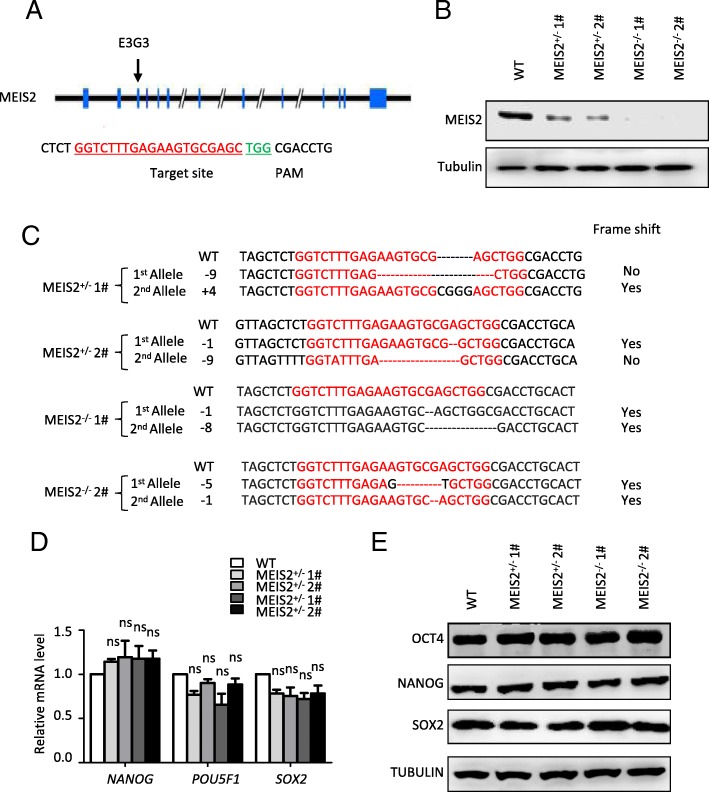
Establishment of MEIS2-deleted hESCs with CRISPR/CAS9 technology. **a** Schematic overview of the small guide RNA-targeting sequence at the MEIS2 locus of human cells. The small guide RNA-targeting sequence is labeled in red, and the protospacer-adjacent motif (PAM) sequence is marked in green. **b** Western blotting analysis of MEIS2 expression in WT, MEIS2^+/−^ hESCs and MEIS2^−/−^ hESCs. Tubulin was used as a loading control. **c** DNA sequencing analysis of MEIS2 locus in MEIS2^+/−^ and MEIS2^−/−^ hESCs. **d** Real-time PCR analysis of pluripotent transcription factors of undifferentiated WT, MEIS2^+/−^, and MEIS2^−/−^ hESCs. RNA relative expression was normalized using ACTB (= 1) in undifferentiated WT cells. Results are shown as means ± SEM (*n* = 3). **e** Western blotting of undifferentiated WT, MEIS2^+/−^, and MEIS2^−/−^ hESCs with antibodies against OCT4, SOX2, and NANOG. Tubulin was used as a loading control. NS not significant

CRIPSR/Cas9-mediated gene deletion provides an effective approach to explore the function of gene in hESC differentiation and therefore facilitates our understanding of human development. To investigate whether MEIS2 has a function in hematopoietic differentiation, we created MEIS2-deficient H1 hESCs using the CRIPSR/Cas9 technology. A small guide RNA (sgRNA) targeting *MEIS2* exon3 was designed, and its genome editing efficacy was validated by the use of surveyor assay (Fig. 2a and Additional file 1: Figure S1A). After expanding H1 hESCs clonally with a sgRNA-targeting *MEIS2* exon3, we successfully generated two H1 clones with homozygous MEIS2 deletion, as accessed with direct sequencing analysis (Fig. 2b, c). Additionally, two H1 clones with MEIS2 heterozygous deletion were included in parallel with the MEIS2 homozygous clones for subsequent studies. The H1 clones with MEIS2 heterozygous or homozygous deletion exhibited compact morphologies and high-level expression of pluripotency markers (NANOG, OCT4, and SOX2) comparable with that of wild-type cells, as accessed with real-time PCR, western blotting, and immunofluorescence studies (Fig. 2d, e and Additional file 1: Figure S1B), suggesting that MEIS2 deletion does not alter pluripotency.

**Fig. 3:**
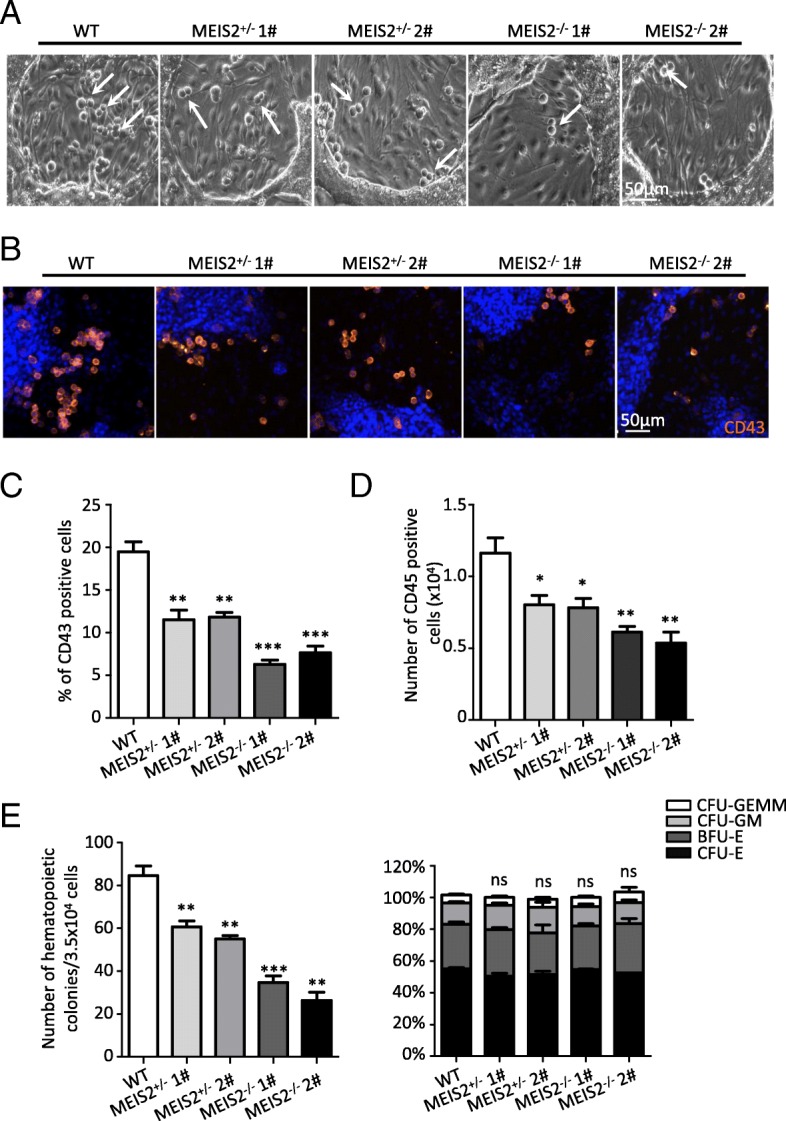
MEIS2 deletion impairs early hematopoiesis of hESCs. **a** Representative morphology of “cobblestone-like” cells generated from WT, MEIS2^+/−^, and MEIS2^−/−^ hESCs at day 7 of hematopoietic differentiation. The “cobblestone-like” cells were indicated by orange arrows. Scale bar, 50 μm. **b** Immunofluorescence of WT, MEIS2^+/−^, and MEIS2^−/−^ hESCs with antibody against CD43 (red) at day 7 of hematopoietic differentiation. Nuclei were stained with DAPI (blue). Scale bars, 50 μm. **c** Flow cytometry analysis of CD43^+^ hematopoietic precursors generated from WT, MEIS2^+/−^, and MEIS2^−/−^ hESCs at day 7 of differentiation. **d** Flow cytometry analysis of CD45^+^ hematopoietic cells generated from WT, MEIS2^+/−^, and MEIS2^−/−^ hESCs at day 11 of differentiation. **e** The total number of colonies derived from WT, MEIS2^+/−^, and MEIS2^−/−^ hESCs (left). The proportion of BFU-E, CFU-E, CFU-GM, and CFU-GEMM in total colonies. Results are shown as means ± SEM (*n* = 3). NS not significant; *P < 0.05, **P < 0.01, and ***P < 0.001

To evaluate the effect of MEIS2 deletion on hematopoietic differentiation of hESCs, we differentiated WT, *MEIS2*^+/−^, and *MEIS2*^−/−^ H1 hESCs under the CDS condition. MEIS2 deletion severely impaired the generation of cobblestone-like cells (Fig. 3a). There was a substantial reduction in the total percentage of CD43^+^ HPCs derived from *MEIS2*^−/−^ H1 hESCs when compared with the wild-type cells, as shown by immunofluorescence and flow cytometry analysis (Fig. 3b, c). Furthermore, the induction of CD45^+^ hematopoietic cells, which arise from CD43^+^ HPCs, was also profoundly suppressed by MEIS2 deletion (Fig. 3d). We further confirmed the decreased generation of functional hematopoietic cells from *MEIS2*^−/−^ hESCs using colony-forming unit (CFU) assay. Interestingly, there was little difference in the proportion of different colony types (BFU-E, CFU-E, CFU-GM, and CFU-GEMM) (Fig. 3e, Additional file 2: Figure S2A), suggesting that MEIS2 deletion had minimal effects on the differentiation potential of HPCs derived from hESCs. The decrease in hematopoietic cell generation was also observed in MEIS2 heterozygous cells. Thus, MEIS2 deletion impairs early hematopoietic differentiation from hESCs.

### MEIS2 deletion suppresses HEP specification and EHT

The impairment of hematopoietic differentiation caused by MEIS2 deletion may result from at least three possibilities: (i) increased proliferation or decreased apoptosis of hematopoietic cells, (ii) suppressed generation of hematopoietic cell precursors, and (iii) impairment of EHT. To distinguish these possibilities, we first examined the effect of MEIS2 deletion on the proliferation and apoptosis of CD43^+^ hematopoietic cells using Ki67 incorporation and annexin V staining assays, respectively. As shown in Additional file 3: Figure S3A-B, there was no significant difference in the fraction of cycling or apoptotic cells between CD43^+^ hematopoietic cells derived from *MEIS2*^+/−^ or *MEIS2*^*−/−*^ hESCs and from the wild-type hESCs.

**Fig. 4:**
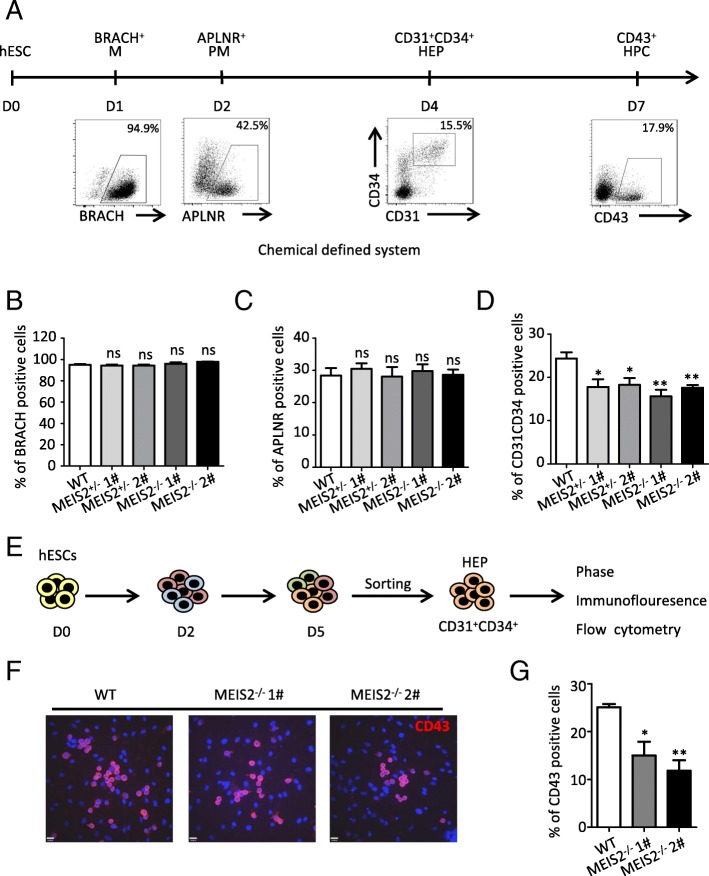
MEIS2 regulates HEP specification and EHT of human early hematopoiesis by targeting TAL1. **a** Schematic overview of hESC hematopoietic differentiation in chemical defined system. M mesoderm, LM lateral mesoderm, HEP hemogenic endothelial progenitors, HPC hematopoietic progenitor cells. **b**–**d** Flow cytometry analysis of BRACH^+^ mesoderm cells (**b**), APLNR^+^ lateral mesoderm cells (**c**), and CD31^+^CD34^+^ hemogenic endothelial progenitors (**d**) generated from WT, MEIS2^+/−^, and MEIS2^−/−^ hESCs at day 1, day 2, and day 4 of differentiation, respectively. **e** Strategy of assessing the ability of CD31^+^CD34^+^ hemogenic endothelial progenitors to generate CD43^+^ hematopoietic precursors. The emerging CD31^+^CD34^+^ cells generated from WT or MEIS2^−/−^ hESCs at day 4 were isolated and re-plated into differentiation condition with 40 ng/ml VEGF and 50 ng/ml bFGF and 20 μM SB431542 for 72 h, analyzed with flow cytometry. **f** Immunofluorescence of CD43^+^ (red) hematopoietic precursors generated from isolated CD31^+^CD34^+^ hemogenic endothelial progenitors. Nuclei were stained with DAPI (blue). **g** Flow cytometry analysis of CD43^+^ hematopoietic progenitors derived from CD31^+^CD34^+^ cells. Results are shown as means ± SEM (*n* = 3). NS not significant; *P < 0.05, **P < 0.01, and ***P < 0.001

The entire hematopoietic differentiation process from H1 hESCs can be monitored in our differentiation model (Fig. 4a). We therefore evaluated the impact of MEIS2 deletion on the generation of hematopoietic precursor cells from H1 hESCs. No significant changes in the fraction of brachyury^+^ mesoderm cells and APLNR^+^ lateral mesoderm cells were observed with MEIS2 deletion (Fig. 4b, c). In contrast, MEIS2 deletion profoundly reduced the population of CD31^+^CD34^+^ HEPs, suggesting an important role of MEIS2 in HEP specification (Fig. 4d). Furthermore, as shown in Additional file 3: Figure S3C-D,there was no significant difference in the fraction of proliferation or apoptotic cells in APLNR^+^ mesoderms and CD31^+^CD34^+^ HEPs derived from *MEIS2*^+/−^ or *MEIS2*^*−/−*^ hESCs and from the wild-type hESCs. EHT is a vital step of hematopoietic cell generation. To test the hypothesis that MEIS2 may play a role in this process, we enriched CD31^+^CD34^+^ HEPs derived from wild-type and MEIS2-deleted H1 hESCs at day 4 of differentiation and measured their hematopoietic differentiation potential in culture medium containing VEGF, bFGF, and SB431542 (Fig. 4e). Remarkably, the generation of cobblestone-like cell from MEIS2-deleted hESCs was impaired in comparison with the wild-type cells (Additional file 3: Figure S3E). Immunofluorescence and flow cytometry analysis further showed that MEIS2 deletion significantly inhibited the induction of CD43^+^ HPCs from CD31^+^CD34^+^ HEPs (Fig. 4f, g), confirming the impairment of EHT. In contrast, MEIS2 deletion had little effect on the endothelium differentiation potential of CD31^+^CD34^+^ HEPs, as evidenced by results from flow cytometer analysis, the acetylated low-density lipoprotein (AcLDL) uptake assay and the tube formation assay (Additional file 3: Figure S3F), confirming that MEIS2 deletion specifically inhibits the hematopoietic differentiation potential of HEPs. Together, our findings demonstrated that MEIS2 deletion impairs hESC hematopoietic differentiation by suppressing HEP specification and EHT.

### MEIS2 controls HEP specification and EHT by targeting TAL1

**Fig. 5:**
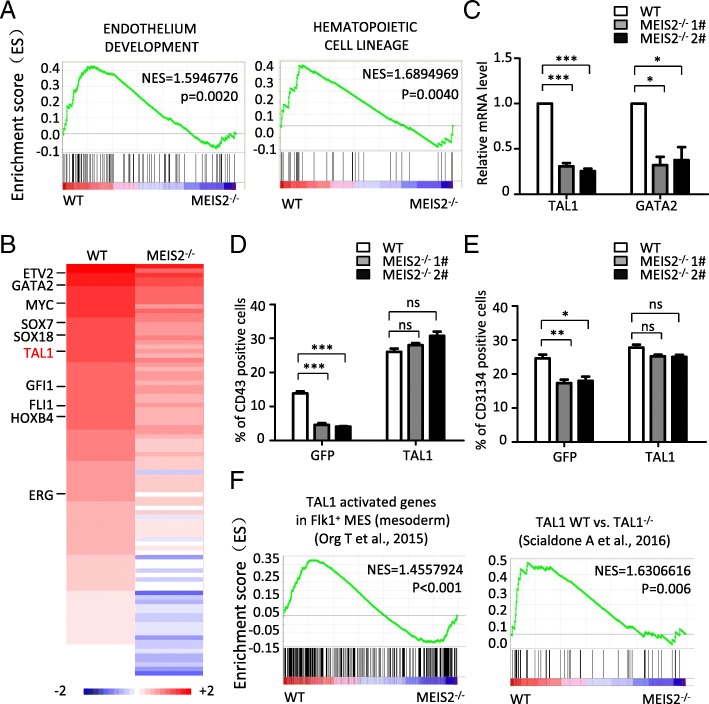
MEIS2 deletion impairs early hematopoietic differentiation from hESCs by suppressing HEP specification and EHT. **a** GSEA depicting the enriched processes in “endothelium development” and “hematopoietic cell lineages.” **b** Heatmap of 93 downregulated genes in differentiated cells from MEIS2^−/−^ hESCs compared with WT. Endothelium specification and hematopoiesis associated genes are listed on the right. **c** Expression of GATA2 and TAL1 in differentiated cells from WT and MEIS2^−/−^ hESCs at day 5 of differentiation with real-time PCR analysis. **d** Flow cytometry analysis of CD43^+^ hematopoietic precursors generated from WT and MEIS2^−/−^ hESCs at day 7 of differentiation with or without exogenous expression of TAL1. The analysis was performed by gating GFP^+^ cells. **e** Flow cytometry analysis of CD31^+^ CD34^+^ HEPs generated from WT and MEIS2^−/−^ hESCs at day 5 of differentiation with or without exogenous expression of TAL1. The analysis was performed by gating GFP^+^ cells. **f** GSEA profiles show the enrichment of genes expressed differentially between WT and MEIS2^−/−^ hESCs and those expressed differentially in “TAL1-activated genes in FLK+ MES (mesoderm)” (left) and TAL1 WT vs TAL1^−/−^ gene sets (right). Results are shown as means ± SEM (*n* = 3). NS not significant; *P < 0.05, **P < 0.01, and ***P < 0.001

To investigate the molecular mechanism by which MEIS2 regulates hESC hematopoietic differentiation, we performed RNA-Seq using the differentiated cells with or without MEIS2 deletion. Endothelium development and hematopoietic fate decision-related genes were significantly enriched in WT cells compared with MEIS2-deleted cells, consistent with the impairment of HEP generation and EHT caused by MEIS2 deletion (Fig. 5a). To identify the target genes mediating the action of MEIS2, we further analyze the transcriptional factors downregulated by MEIS2 deletion. As shown in Fig. 5b, 93 genes were significantly downregulated in MEIS2-deleted cell when compared with WT cells, including GATA2, TAL1, and GFI1, all of which have been reported to be crucial for vertebrate hematopoietic development [12, 13, 30]. Among these, TAL1 and GATA2 were especially attractive due to their important roles in human hematopoiesis. The suppressed expression of TAL1 and GATA2 induced by MEIS2 deletion was first validated by real-time PCR analysis (Fig. 5c). Subsequently, we tested whether enforced expression of TAL1 or GATA2 could rescue the defect of hematopoietic differentiation caused by MEIS2 deletion. Ectopic expression of *TAL1* or *GATA2* was confirmed by western blot analysis (Additional file 4: Figure S4A). Remarkably, *TAL1* overexpression nearly completely reversed the decrease in CD43^+^ HPCs caused by MEIS2 deletion (Fig. 5d). However, GATA2 overexpression failed to rescue this defect (Additional file 4: Figure S4B). Furthermore, the defect of CD31^+^CD34^+^ HEP generation was also reversed by TAL1 overexpression (Fig. 5e), further suggesting that TAL1 mediates the function of MEIS2 during hESC hematopoietic differentiation. To further confirm that TAL1 functions downstream of MEIS2 during hematopoiesis, we used GSEA to compare potential gene expression overlaps between WT cells vs MEIS2-deleted cells and previously published gene sets regulated by Tal1 in vitro and in vivo. During the specification from mesodermal cells to hemogenic endothelium, 592 genes were found to be upregulated upon Tal1 overexpression in mouse ES cells [31]. We performed GSEA using the gene sets and found that the altered molecular signature by MEIS2 deletion was also enriched in the upregulated gene set caused by Tal1 overexpression (Fig. 5f). Tal1-null endothelium failed to transit to blood cells during mouse embryonic hematopoiesis. By using single-cell expression profiling, Scialdone et al. recently identified 50 genes specifically downregulated in embryonic endothelial cells with Tal1 deletion [32]. GSEA analysis revealed strong overlaps between genes repressed by MEIS2 deletion in hESC hematopoietic differentiation and the 50 genes (Fig. 5g), further supporting the notion that TAL1 functions as a downstream target of MEIS2 during early hematopoietic differentiation. Thus, MEIS2 controls HEP specification and EHT by targeting TAL1.

### MEIS2 is dispensable for human megakaryopoiesis and thrombopoiesis

**Fig. 6:**
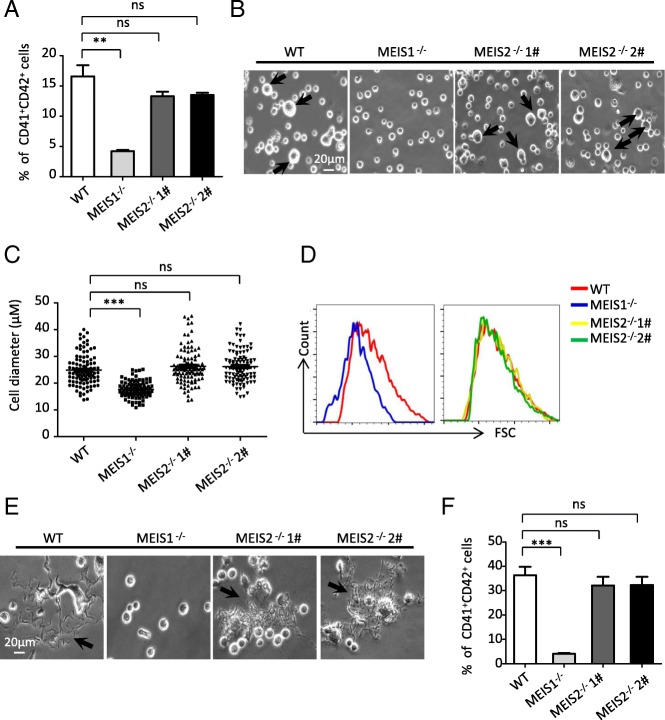
MEIS2 is dispensable for megakaryopoiesis and thrombopoiesis. **a** Representative morphologies of differentiated WT and MEIS2^−/−^ hESCs at day 3 of megakaryocytic differentiation. Large cells are indicated by orange arrows. Scale bar, 20 μm. **b** Flow cytometry analysis of CD41a^+^CD42b^+^ megakaryocytes at day 3 of megakaryocytic differentiation. **c** Distribution of diameter of 100 cells in each group. The sizes of cells were measured by using Nano software. **d** Flow cytometry analysis of the cell size in WT and MEIS2^−/−^ hESCs at day 3 of megakaryocytic differentiation. **e** Representative morphologies of proplatelets generated from WT and MEIS2^−/−^ hESCs at day 6 of megakaryocytic differentiation. Scale bar, 20 μm. **f** Flow cytometry analysis of CD41a^+^CD42b^+^ proplatelets at day 6 of megakaryocytic differentiation. MEIS1^−/−^ hESCs were used as a control. Results are shown as means ± SEM (*n* = 3). NS not significant; *P < 0.05, **P < 0.01, and ***P < 0.001

A previous study demonstrated that *Meis2-*deficient mice exhibit hemorrhaging phenotype and die between E13.5 and E14.5 [20], suggesting that *Meis2* may be crucial for megakaryopoiesis and thrombopoiesis. Thus, we asked whether *MEIS2* deletion disrupts megakaryocytic specification and platelet production from hESCs. We recently depicted an essential role of MEIS1 in megakaryopoiesis and thrombopoiesis of hESCs [27]. Therefore, *MEIS1-*deficient H1 hESCs were included as a control in our following studies. By applying the megakaryocytic differentiation model previously reported by us, we first determined the effect of *MEIS2* deletion on megakaryocytic differentiation. In contrast to the robust decrease in the generation of CD41a^+^CD42b^+^ megakaryocytes caused by *MEIS1* deletion, *MEIS2* deletion did not significantly alter the fraction of this population (Fig. 6a). The increase in cell size is the hallmark of megakaryocytic differentiation and maturation. Thus, we next analyzed the impact of *MEIS2* deletion on the cell volume by using cell diameter quantification and flow cytometry analysis. As shown in Fig. 6b–d, there was little difference in the cell diameter of differentiated cells from H1 hESCs with or without MEIS2 deletion. Unlike the deficiency observed in megakaryocytes with MEIS1 deletion, MEIS2-deficient megakaryocytes could form normal proplatelets (Fig. 6e). Moreover, no significant changes in the percentages of CD41a^+^CD42b^+^ platelet-sized particles (PLPs) were observed in MEIS2-deficient cells (Fig. 6f). Thus, MEIS2 is dispensable for human megakaryopoiesis and thrombopoiesis.

## Discussion

Elucidating the molecular mechanism governing hematopoietic lineage specification of hESCs will facilitate the generation of HSCs with long-term transplantation capacity. In the present study, we identified MEIS2 as a potential regulator of early human hematopoiesis. MEIS2 deletion inhibits hematopoietic differentiation of hESCs. Furthermore, we found that suppression of both HEP specification and EHT leads to the impairment of HPC generation. TAL1 mediates the action of MEIS2 in early human hematopoiesis. Interestingly, unlike MEIS1, MEIS2 is dispensable for megakaryopoiesis and thrombopoiesis from hESCs. Therefore, our findings not only deepen our understanding of human hematopoiesis and megakaryopoiesis but may also offer novel insights to facilitate the production of functional HSCs and other types of blood cells from hPSCs for cell-based therapies.

Meis2 knockout mice display smaller livers and severe anemia [20], suggesting that Meis2 may play a role in mouse embryonic hematopoiesis. However, the impact of MEIS2 deletion on human hematopoiesis remains unknown. By taking advantage of a hESC-based hematopoietic differentiation model, we found that endogenous MEIS2 expression is in parallel with hESC hematopoietic specification and is at high levels in the HEPs and CD43^+^ blood cells. Further functional studies demonstrated that MEIS2 deletion impairs the generation of HPCs from hESCs, confirming its critical role in early human hematopoiesis. Interestingly, MEIS2 deletion does not affect proliferation and survival of hESC-derived hematopoietic cells, in disagreement with the finding that enforced expression of Meis2 promotes mouse ESC hematopoietic differentiation by maintaining proliferation of hematopoietic progenitors [26]. We speculate that the discrepancy between the hESC and the mouse ESC studies may be attributed to species-specific functions of MEIS2 or the difference of experimental techniques: loss-of-function analysis was used for hESCs while gain-of-function studies were conducted for mouse ESCs. Consistent with the substantial decrease of MEIS2 expression in CD45^+^ cells differentiated from CD43^+^ cells, MEIS2 deletion has little effect on the differentiation potential of hematopoietic progenitors, as shown by experiments with the CFU assay. These data suggest that MEIS2 is crucial for the induction of human hematopoietic progenitors but may be indispensable for their proliferation and differentiation into mature blood cells.

hESC hematopoietic differentiation recapitulates embryonic hematopoiesis and goes through the processes of mesoderm induction, lateral plate mesoderm specification, HEP emergence, and EHT [7]. Our data demonstrate that MEIS2 deletion impairs hematopoietic differentiation by specifically suppressing HEP generation and EHT instead of induction and lateralization of mesoderm. As the key step of hematopoietic fate decision, EHT is regulated by transcriptional factors in a highly precise pattern during hematopoiesis. RUNX1, GATA2, TAL1, and HOXA9 have been implicated in the regulation of EHT [11–13, 33]. Combining one or more of these genes with other hematopoiesis-related genes is sufficient to directly convert hESCs or human endothelial cells into hematopoietic cells, highlighting the importance of identifying novel regulators of EHT. Therefore, it will be of great interest to explore whether MEIS2 could replace one or more genes to program hESCs or human endothelial cells into hematopoietic cells in further studies.

To understand the molecular mechanism by which MEIS2 regulates human hematopoiesis, we performed gene expression profiling of differentiated cells with or without MEIS2 deletion. As expected, MEIS2 deletion suppresses the molecular signature related to “endothelium development” and “hematopoietic transition from endothelial cells.” Combining gene expression profiling analysis with rescue experiments, we identified and confirmed TAL1 as the target gene mediating the function of MEIS2. Scl/Tal1 is widely accepted as a key factor controlling hematopoietic cell emergence [34]. Tal1 knockout experiments reveal a vital role for Tal1 in invertebrate and mouse embryonic hematopoiesis [35]. By using hESCs as a human developmental model, Real et al. reported TAL1 as a master regulator of human hematopoiesis [13]. Similar to MEIS2, TAL1 regulates hematopoietic differentiation by controlling HEP specification and subsequent hematopoietic commitment. Although the role of TAL1 in human hematopoiesis is well characterized, its regulatory mechanism remains largely unknown. Our studies reveal, for the first time, that MEIS2 acts upstream of TAL1 during human hematopoiesis. The detailed mechanism underlying MEIS2 regulation of TAL1 expression during human hematopoiesis awaits future investigation.

The strong hemorrhaging phenotype in *Meis2-*deficient mice led us to explore the impact of MEIS2 deletion on human megakaryopoiesis and thrombopoiesis [20]. Unexpectedly, we found that MEIS2-deleted HPCs can normally differentiate into megakaryocytes and generate platelets. The results differ from earlier reports that *Meis2* promotes megakaryocyte lineage specification while suppressing erythroid progenitor differentiation in mouse ESCs [26]. The discrepancy between the human and the mouse studies may reflect the distinct function of MEIS2 in the two species. Similar species-specific functions of other genes have been reported previously. For example, Zhang et al. showed that PAX6 is essential for neuroectoderm cell fate decision in hESCs but is not required for mouse neuroectoderm specification [36]. Therefore, our data reiterate the distinction between humans and mice, further supporting hESCs as a unique model for deciphering the molecular basis regulating human development.

## Conclusions

In this study, by using whole-genome gene profiling, we identified Myeloid Ectopic Viral Integration Site 2 homolog (MEIS2) as a potential regulator of hESC early hematopoietic differentiation. Furthermore, our data reveal that MEIS2 deletion inhibits hemogenic endothelial cell (HEP) specification and endothelial to hematopoietic transition (EHT). Mechanistically, TAL1 acts as the downstream gene mediating the function of MEIS2 during early hematopoiesis. Interestingly, unlike MEIS1, MEIS2 is dispensable for human megakaryopoiesis and thrombocytopoiesis, although mice lacking Meis2 show severe hemorrhaging defects.

Together, our findings advance the understanding of human hematopoietic development, and this represents important contributions to the mechanistic understanding of hPSC hematopoietic differentiation, especially during HEP specification and EHT.

## Additional files

Additional file 1: **Figure S1.** Targeted deletion of MEIS2 in human hESCs. (A) Surveyor assay of sgMESI2-E3G3-mediated cleavage at MEIS2 loci in H1 hESCs. (B) Immunofluorescence analysis of OCT4, SOX2, and NANOG in undifferentiated WT, MEIS2^+/−^, and MEIS2^−/−^ hESCs. Scale bar, 80 μm. (PDF 136 kb)

Additional file 2: **Figure S2.** MEIS2 deletion impairs early hematopoietic differentiation of hESCs. (A) Representative morphology of BFU-E, CFU-GM, and CFU-GEMM derived from WT. (PDF 84 kb)

Additional file 3: **Figure S3.** MEIS2 deletion suppresses endothelium specification and EHT. (A) Proliferation of CD43^+^ HPCs derived from WT, MEIS2^+/−^, and MEIS2^−/−^ hESCs was analyzed by Ki67. (B) Apoptosis of CD43^+^ HPCs derived from WT, MEIS2^+/−^, and MEIS2^−/−^ hESCs was determined with annexin V. (C) Proliferation and apoptosis of APLNR^+^ mesoderm derived from WT, MEIS2^+/−^, and MEIS2^−/−^ hESCs analyzed by Ki67 and annexin V respectively. (D) Proliferation and apoptosis of CD31^+^CD34^+^ HEPs derived from WT, MEIS2^+/−^, and MEIS2^−/−^ hESCs analyzed by Ki67 and annexin V respectively. (E) Representative morphologies of “cobblestone-like” cells differentiated from WT, MEIS2^+/−^, and MEIS2^−/−^ hESCs during EHT. Scale bar, 80 μm. (F) The endothelial potential of CD31^+^CD34^+^ cells derived from WT, MEIS2^+/−^, and MEIS2^−/−^ hESCs. Isolated CD31^+^CD34^+^ cells were cultured in endothelial condition with subsequent analyses including AcLDL uptake, tube formation, and flow cytometry. Results are shown as means ± SEM (*n* = 3).NS, not significant. (PDF 781 kb)

Additional file 4: **Figure S4.** MEIS2 deletion suppresses hematopoietic differentiation by targeting TAL1. (A) Western blotting analysis of expression of TAL1 and GATA2 proteins (both FLAG-tagged) in differentiated cells from WT and MEIS2^−/−^ hESCs using lentivirus infection. Vector with GFP only was used as a control. (B) Flow cytometry analysis of CD43^+^ hematopoietic precursors generated from WT and MEIS2^−/−^ hESCs at day 7 of differentiation with or without exogenous expression of GATA2. Results are shown as means ± SEM (*n* = 3). NS, not significant, *P < 0.05, **P < 0.01, and ***P < 0.001. (PDF 59 kb)

## Abbreviations

EHT: Endothelial to hematopoietic transition
HEP: Hemogenic endothelial cell
HPCs: Hematopoietic progenitor cells
MEIS2: Myeloid Ectopic Viral Integration Site 2 homolog
