# Correction to: MEIS2 regulates endothelial to hematopoietic transition of human embryonic stem cells by targeting TAL1

**DOI:** 10.1186/s13287-020-01913-2

**Published:** 2020-10-19

**Authors:** Mengge Wang, Hongtao Wang, Yuqi Wen, Xiaoyuan Chen, Xin Liu, Jie Gao, Pei Su, Yuanfu Xu, Wen Zhou, Lihong Shi, Jiaxi Zhou

**Affiliations:** 1 State Key Laboratory of Experimental Hematology, Institute of Hematology and Blood Diseases Hospital, Tianjin, 300020 China; 2 Center for Stem Cell Medicine, Chinese Academy of Medical Sciences and Department of Stem Cells and Regenerative Medicine, Peking Union Medical College, Tianjin, 300020 China; 3 School of Basic Medical Science and Cancer Research Institute, Central South University, Changsha, 410013 China

**Correction to: Stem Cell Res Ther 9, 340 (2018)**

**https://doi.org/10.1186/s13287-018-1074-z**

Following publication of the original article [1], the authors identified an editing error in Additional file 1: Fig. S1. They put the same picture in NANOG (MEIS2+/− 1#) and SOX2 (MEIS2+/− 2#) by mistake when they assembled the Fig. S1B. The heterozygous 1# and 2#, and homozygous 1# and 2# have been renamed as well.

The correct figure is given below.

**Fig. 1:**
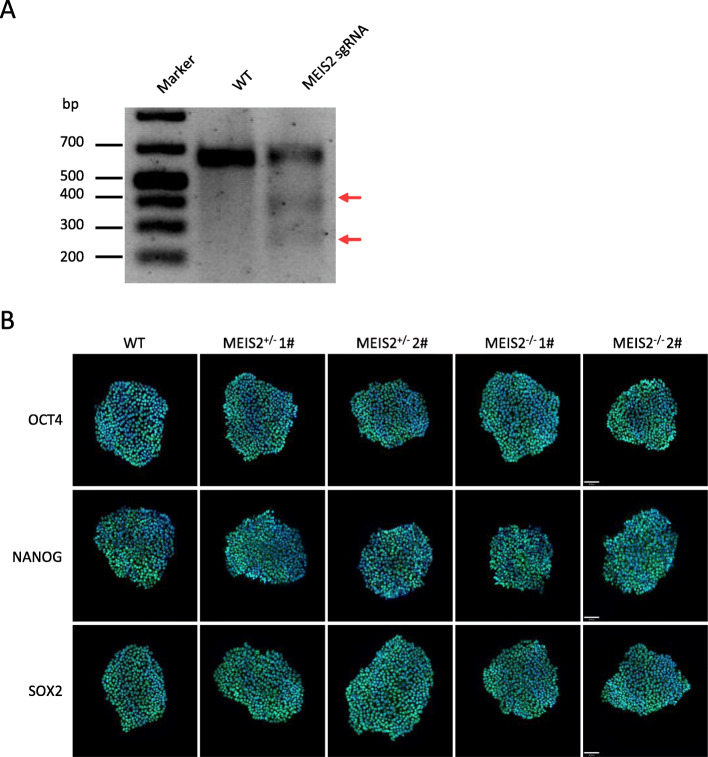
Targeted deletion of MEIS2 in human hESCs. (A) Surveyor assay of sgMESI2-E3G3-mediated cleavage at MEIS2 loci in H1 hESCs. (B) Immunofluorescence analysis of OCT4, SOX2, and NANOG in undifferentiated WT, MEIS2^+/−^, and MEIS2^−/−^ hESCs. Scale bar, 80 μm.
