# Supplementary figures and images for: MEIS2 regulates endothelial to hematopoietic transition of human embryonic stem cells by targeting TAL1
Source: Stem Cell Res Ther. 2018 Dec 7;9:340. doi: 10.1186/s13287-018-1074-z (PMC6286587; doi:10.1186/s13287-018-1074-z)

A

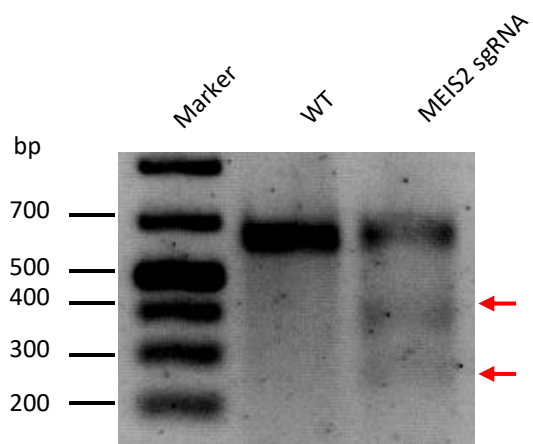

B

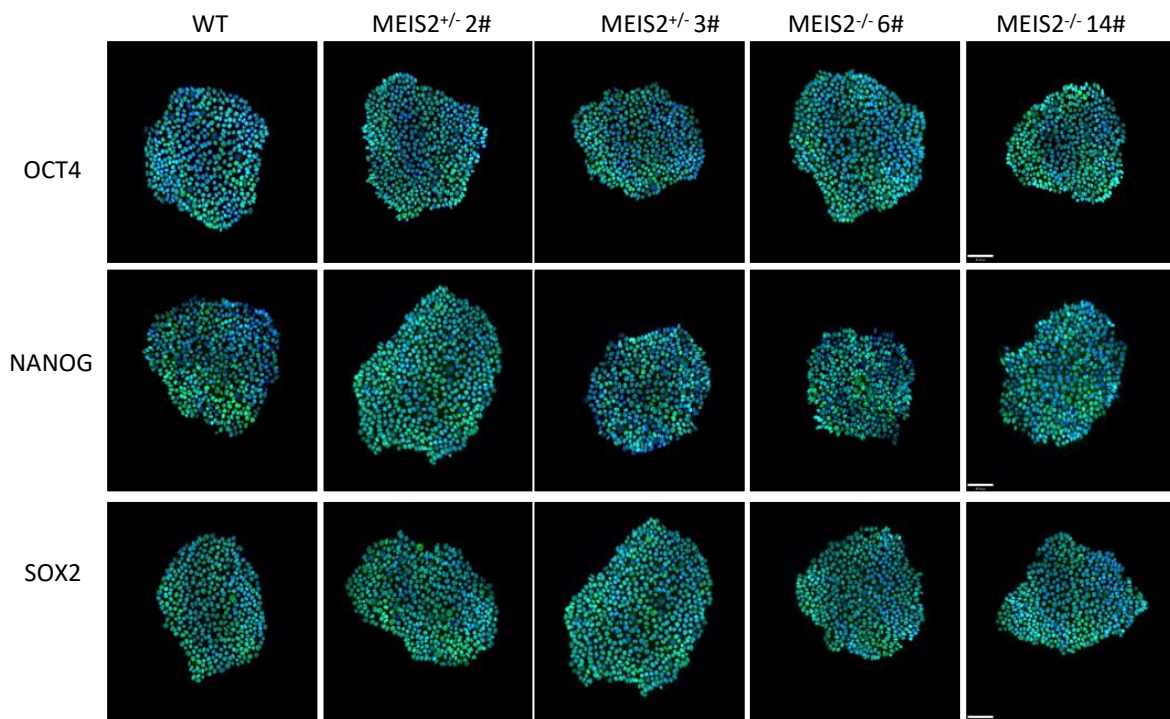

Supplement: Supplementary file 1 — Figure S1. Targeted deletion of MEIS2 in human hESCs. (A) Surveyor assay of sgMESI2-E3G3-mediated cleavage at MEIS2 loci in H1 hESCs. (B) Immunofluorescence analysis of OCT4, SOX2, and NANOG in undifferentiated WT, MEIS2+/−, and MEIS2−/− hESCs. Scale bar, 80 μm. (PDF 136 kb) [file 13287_2018_1074_MOESM1_ESM.pdf]

A

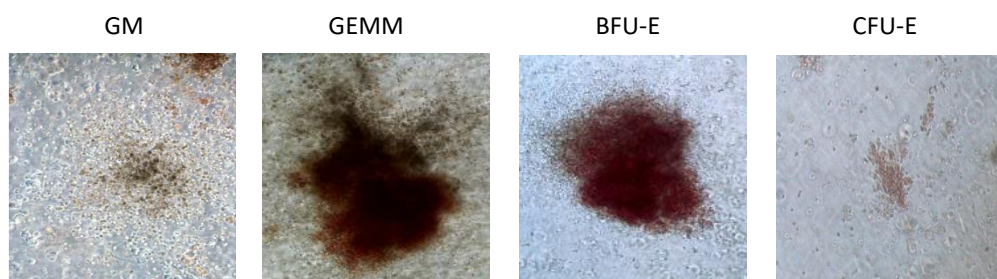

Supplement: Supplementary file 2 — Figure S2. MEIS2 deletion impairs early hematopoietic differentiation of hESCs. (A) Representative morphology of BFU-E, CFU-GM, and CFU-GEMM derived from WT. (PDF 84 kb) [file 13287_2018_1074_MOESM2_ESM.pdf]

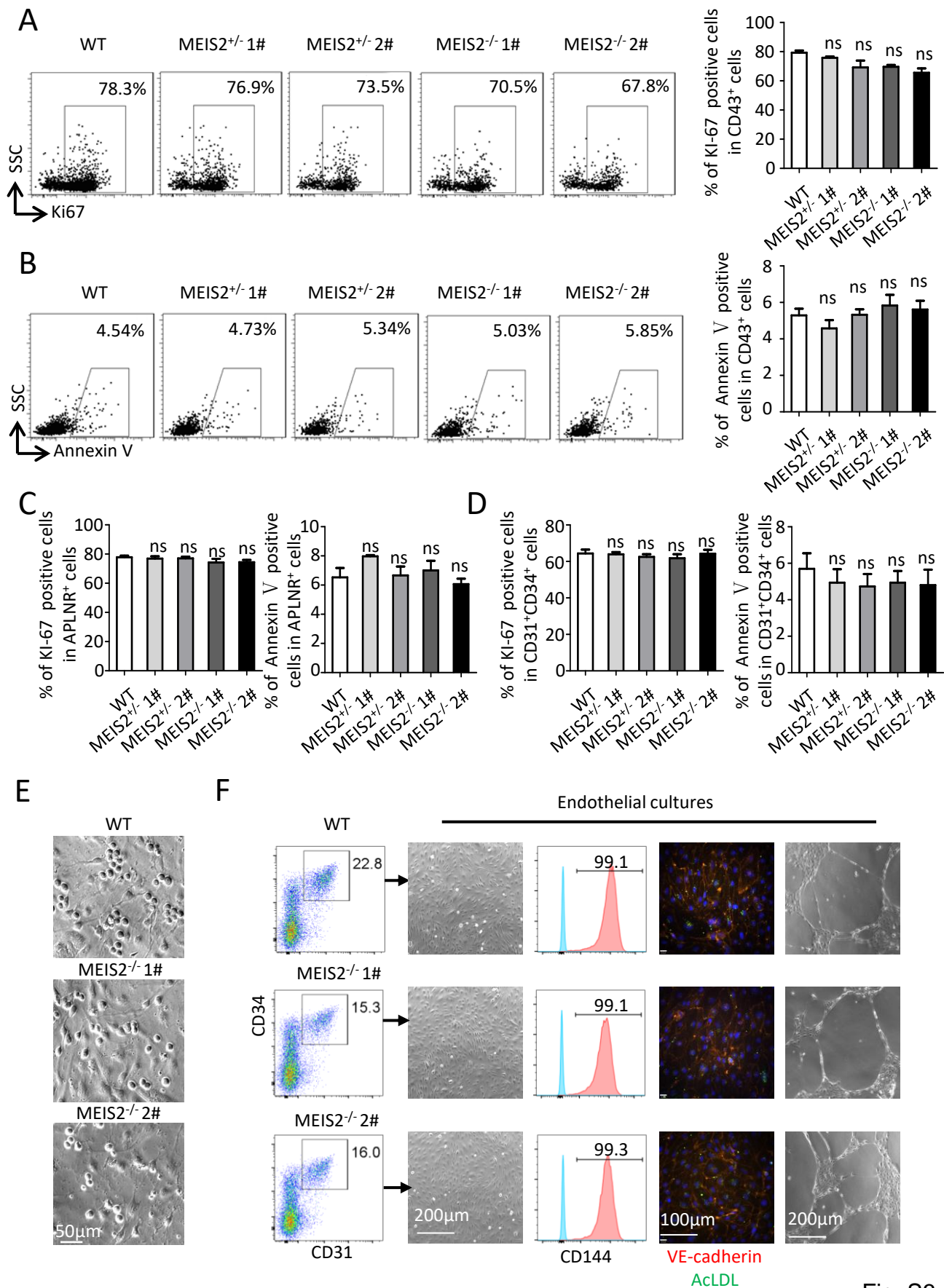

Fig. S3

Supplement: Supplementary file 3 — Figure S3. MEIS2 deletion suppresses endothelium specification and EHT. (A) Proliferation of CD43+ HPCs derived from WT, MEIS2+/−, and MEIS2−/− hESCs was analyzed by Ki67. (B) Apoptosis of CD43+ HPCs derived from WT, MEIS2+/−, and MEIS2−/− hESCs was determined with annexin V. (C) Proliferation and apoptosis of APLNR+ mesoderm derived from WT, MEIS2+/−, and MEIS2−/− hESCs analyzed by Ki67 and annexin V respectively. (D) Proliferation and apoptosis of CD31+CD34+ HEPs derived from WT, MEIS2+/−, and MEIS2−/− hESCs analyzed by Ki67 and annexin V respectively. (E) Representative morphologies of “cobblestone-like” cells differentiated from WT, MEIS2+/−, and MEIS2−/− hESCs during EHT. Scale bar, 80 μm. (F) The endothelial potential of CD31+CD34+ cells derived from WT, MEIS2+/−, and MEIS2−/− hESCs. Isolated CD31+CD34+ cells were cultured in endothelial condition with subsequent analyses including AcLDL uptake, tube formation, and flow cytometry. Results are shown as means ± SEM (n = 3).NS, not significant. (PDF 781 kb) [file 13287_2018_1074_MOESM3_ESM.pdf]

A

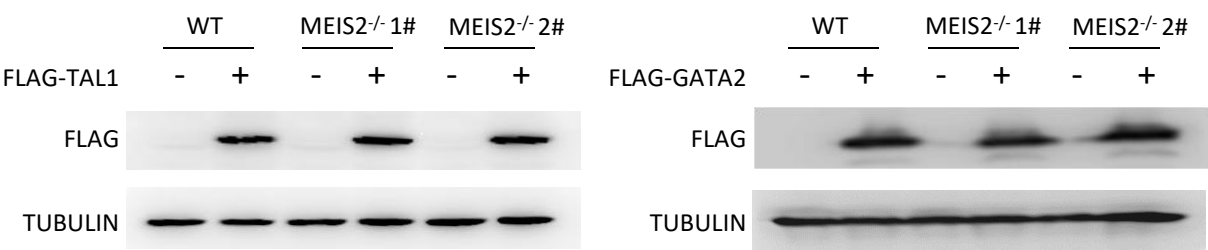

B

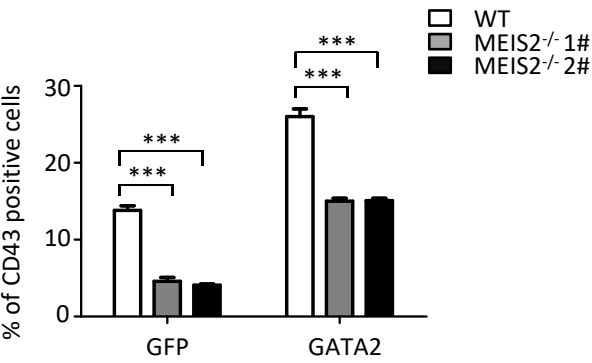

Fig. S4

Supplement: Supplementary file 4 — Figure S4. MEIS2 deletion suppresses hematopoietic differentiation by targeting TAL1. (A) Western blotting analysis of expression of TAL1 and GATA2 proteins (both FLAG-tagged) in differentiated cells from WT and MEIS2−/− hESCs using lentivirus infection. Vector with GFP only was used as a control. (B) Flow cytometry analysis of CD43+ hematopoietic precursors generated from WT and MEIS2−/− hESCs at day 7 of differentiation with or without exogenous expression of GATA2. Results are shown as means ± SEM (n = 3). NS, not significant, *P < 0.05, **P < 0.01, and ***P < 0.001. (PDF 59 kb) [file 13287_2018_1074_MOESM4_ESM.pdf]
